# Genomic Profiling of Estrogen-Related Receptor Identifies Adult Adipocyte-Specific Targets in *Drosophila*

**DOI:** 10.64898/2026.07.29.741485

**Published:** 2026-07-30

**Authors:** Jun Yin, Douglas B. Rusch, Jitika S. Bhatta, David M. Merritt, Lesley N. Weaver

## Abstract

Adipose tissue is a key metabolic organ for carbohydrate and lipid metabolism. Multiple nutrient sensing pathways and metabolic enzymes operate in adipocytes to control energy production and lipid mobilization in response to physiological conditions. Important regulators of glycolytic and lipid metabolism in many species are members of the estrogen-related receptor (ERR) family of nuclear receptors. We previously showed that ERR is required for transcriptional regulation of glycolytic and pentose phosphate pathway enzymes in adult *Drosophila* females, consistent with what is observed in *ERR* mutant males and larvae. However, the cell-type specific targets of ERR in adipose and other tissues have not been fully elucidated. Here, using a modified targeted DamID approach (NanoDam), we identified ERR occupancy specifically in adult adipocytes at the loci that encode genes involved in glycolysis, the pentose phosphate pathway, and fatty acid metabolism. Overall, our results predict that ERR centrally functions in adipose tissue to regulate multiple metabolic pathways.

## Full Text Availability

The license terms selected by the author(s) for this preprint version do not permit archiving in PMC. The full text is available from the preprint server.

